# Zolpidem Profoundly Augments Spared Tonic GABA_A_R Signaling in Dentate Granule Cells Ipsilateral to Controlled Cortical Impact Brain Injury in Mice

**DOI:** 10.3389/fnsys.2022.867323

**Published:** 2022-05-26

**Authors:** Jeffery A. Boychuk, Corwin R. Butler, Katalin Cs. Smith, Miklos B. Halmos, Bret N. Smith

**Affiliations:** ^1^Department of Physiology, University of Kentucky, Lexington, KY, United States; ^2^Department of Cellular and Integrative Physiology, UT Health San Antonio, San Antonio, TX, United States; ^3^Department of Anesthesiology and Perioperative Medicine, Oregon Health and Science University, Portland, OR, United States; ^4^Department of Neuroscience, University of Kentucky, Lexington, KY, United States; ^5^Department of Psychology, Georgia State University, Atlanta, GA, United States; ^6^Spinal Cord and Brain Injury Research Center (SCoBIRC), University of Kentucky, Lexington, KY, United States; ^7^Department of Biomedical Sciences, Colorado State University, Fort Collins, CO, United States

**Keywords:** zolpidem, benzodiazepine, GABA_A_ receptors, tonic GABA current, controlled cortical impact (CCI), traumatic brain injury, posttraumatic epilepsy

## Abstract

Type A GABA receptors (GABA_A_Rs) are pentameric combinations of protein subunits that give rise to tonic (I_TonicGABA_) and phasic (i.e., synaptic; I_SynapticGABA_) forms of inhibitory GABA_A_R signaling in the central nervous system. Remodeling and regulation of GABA_A_R protein subunits are implicated in a wide variety of healthy and injury-dependent states, including epilepsy. The present study undertook a detailed analysis of GABA_A_R signaling using whole-cell patch clamp recordings from mouse dentate granule cells (DGCs) in coronal slices containing dorsal hippocampus at 1–2 or 8–13 weeks after a focal, controlled cortical impact (CCI) or sham brain injury. Zolpidem, a benzodiazepine-like positive modulator of GABA_A_Rs, was used to test for changes in GABA_A_R signaling of DGCs due to its selectivity for α_1_ subunit-containing GABA_A_Rs. Electric charge transfer and statistical percent change were analyzed in order to directly compare tonic and phasic GABA_A_R signaling and to account for zolpidem’s ability to modify multiple parameters of GABA_A_R kinetics. We observed that baseline I_TonicGABA_ is preserved at both time-points tested in DGCs ipsilateral to injury (Ipsi-DGCs) compared to DGCs contralateral to injury (Contra-DGCs) or after sham injury (Sham-DGCs). Interestingly, application of zolpidem resulted in modulation of I_TonicGABA_ across groups, with Ipsi-DGCs exhibiting the greatest responsiveness to zolpidem. We also report that the combination of CCI and acute application of zolpidem profoundly augments the proportion of GABA_A_R charge transfer mediated by tonic vs. synaptic currents at both time-points tested, whereas gene expression of GABA_A_R α_1_, α_2_, α_3_, and γ_2_ subunits is unchanged at 8–13 weeks post-injury. Overall, this work highlights the shift toward elevated influence of tonic inhibition in Ipsi-DGCs, the impact of zolpidem on all components of inhibitory control of DGCs, and the sustained nature of these changes in inhibitory tone after CCI injury.

## Highlights

- Dentate granule cells (DGCs) ipsilateral to CCI brain injury exhibit a preserved GABA_A_R-mediated tonic current at 1–2 and 8–13 weeks post-injury.
- DGCs exhibit an unexpected zolpidem-sensitive tonic GABA_A_R current that is markedly enhanced by brain injury (≥84% of controls 1–2 weeks post-injury and ≥75% of controls 8–13 weeks post-injury).
- DGC’s receive more charge transfer from tonic GABA_A_Rs in comparison to synaptic GABA_A_Rs; the ratio of charge transfer is numerically (non-significantly) increased by CCI injury relative to controls (≥103% at 1–2 weeks post-injury and ≥64% at 8–13 weeks post-injury) whereas it is profoundly enhanced by the combination of CCI injury and acute application of zolpidem (≥381% at 1–2 weeks post-injury and ≥251% at 8–13 weeks post-injury).
- Gene expression of GABA_A_R α_1_, α_2_, α_3_, and γ_2_ subunits is unchanged in DG ipsilateral to injury, relative to DG contralateral to injury, 8–13 weeks post-injury.

## Introduction

Neurochemical inhibition of principal cells within the hippocampus is primarily mediated by type A GABA receptors (GABA_A_Rs), that are formed by combinations of α_1–6_, β_1–4_, γ_1–3_, δ, ε, θ, and π subunits (Lüddens and Wisden, 1991; Laurie et al., 1992; Macdonald and Olsen, 1994; Barnard et al., 1998; Whiting et al., 1999). These subunit combinations vary by cell type and result in distinct kinetic profiles and patterns of responses to pharmacological agents (Draguhn et al., 1990; Verdoorn et al., 1990; Davies et al., 1997). Hippocampal dentate granule cells (DGCs) are believed to predominantly express α_1_β_x_γ_2_ and α_4_β_x_δ GABA_A_R subunit combinations; these combinations make well-established contributions to synaptic (i.e., I_SynapticGABA_) and tonic (i.e., I_TonicGABA_) GABA_A_R signaling, respectively (Chang et al., 1996; Kapur and Macdonald, 1999; Mody, 2001; Brown et al., 2002; Stell et al., 2003; Wei et al., 2003; Farrant and Nusser, 2005; Mtchedlishvili and Kapur, 2006; Glykys et al., 2008).

Brain insults including repeated seizures, cerebral ischemia, or brain injury may change the expression or regulation of GABA_A_R subunits to alter functional GABA_A_R currents in DGCs (Gibbs et al., 1997; Brooks-Kayal et al., 1998; Redecker et al., 2002; Leroy et al., 2004; Peng et al., 2004; Sun et al., 2007; Zhang et al., 2007; Kharlamov et al., 2008; Zhan and Nadler, 2009; Pavlov et al., 2011; Gupta et al., 2012; Raible et al., 2012, 2015; Drexel et al., 2015). These changes have often been associated with the coincident loss of local GABAergic interneuron populations following traumatic brain injury (TBI) (Lowenstein et al., 1992; Zhu et al., 1997; Butler et al., 2016, 2017; Frankowski et al., 2019) and subsequent posttraumatic epileptogenesis. However, not all changes in inhibitory tone follow this pattern. Importantly, while synaptic inhibition of DGCs appears reduced after TBI in mouse models of posttraumatic epilepsy, presumably due to loss of inhibitory interneurons, the baseline tonic inhibition of DGCs after brain injury is maintained, even after spontaneous recurrent seizures have developed (Cossart et al., 2001; Pavlov et al., 2011; Boychuk et al., 2016; Butler et al., 2016; Becerra et al., 2021). Additionally, recordings of DGCs from mice with acquired epilepsy after experimental brain injury such as controlled cortical impact (CCI), or after pilocarpine-induced status epilepticus, often require efforts to “unmask” recurrent excitation due to the selective preservation/remodeling of inhibitory circuits (Winokur et al., 2004; Hunt et al., 2009, 2010, 2011). These observations suggest underlying compensatory mechanisms following brain injury to maintain inhibitory tone of DGCs despite a large percentage of local GABAergic interneurons being lost following brain insults.

We have previously reported a preservation of the tonic GABA current in DGCs after CCI that parallels a loss of responsiveness to THIP (Gaboxadol), a GABA_A_R “super-agonist” that exhibits preferential action (i.e., selectivity) for receptor pentamers containing the δ subunit along with a4 and a6 subunits (Saarelainen et al., 2008; Chandra et al., 2010; Boychuk et al., 2016; Butler et al., 2016). These findings using CCI have recently been replicated and expanded upon (Becerra et al., 2021) and suggest remodeling or altered regulation of GABA_A_R subunits, chloride transport, GABA_A_R signaling, and/or other mechanisms. In this study, we focused on the contribution of α1 containing GABA_A_Rs to inhibitory tone of DGCs following TBI injury using the benzodiazepine-like GABA_A_R augmenting agent zolpidem, a potent hypnotic and somnolescent with selectivity for α1 containing GABA_A_Rs and direct clinical implications (Kapur and Macdonald, 1996, 1999; Hollrigel and Soltesz, 1997; Brooks-Kayal et al., 1998; Defazio and Hablitz, 1999; Perrais and Ropert, 1999; Nusser and Mody, 2002; Cohen et al., 2003; Lindquist and Birnir, 2006; Leppä et al., 2011).

Using whole-cell patch-clamp recordings from DGCs *in vitro*, we tested the hypothesis that tonic GABA_A_R signaling is spared in DGCs located ipsilateral to CCI injury (Ipsi-DGCs), since this sparing has previously been observed and parallels a loss of responsiveness to THIP (Boychuk et al., 2016; Butler et al., 2016; Becerra et al., 2021). We also hypothesized that zolpidem sensitivity in GABA_A_R signaling would be reduced after CCI, since this compound is considered a modifier of synaptic α1 containing GABA_A_Rs and phasic signaling to DGCs is corrupted after brain injury. Outcomes of these studies have implications for treatments to alter DGC excitability after brain injury and consequent posttraumatic epileptogenesis.

## Materials and Methods

### Animals

Six- to eight-week-old adult male CD-1 mice (Invigro-Harlan) were housed under a normal 14/10 h light/dark cycle and given food and water *ad libitum*. All animals were acclimated to the University of Kentucky vivarium for at least 1 week prior to experimentation. Male mice were tested in this study because cycling female hormones, e.g., estrogen and progesterone, robustly alter GABA_A_R signaling and require dedicated examination of ovariectomized (OVX) females and OVX-treated females given select hormone replacement and/or pharmacological modification of key biochemical steps in ovarian hormone pathways. This biology has been studied by our labs (Littlejohn and Boychuk, 2021) and others (Twyman and Macdonald, 1992; Fáncsik et al., 2000; Wohlfarth et al., 2002; Griffiths and Lovick, 2005; Maguire and Mody, 2007; Abramian et al., 2014; Carver et al., 2014). All procedures were approved by the University of Kentucky Animal Care and Use Committee (Assurance #A3336-01) and adhered to NIH guidelines for the care and use of laboratory animals.

### Brain Injury

Mice were administered a unilateral, focal contusion injury using CCI as previously reported (Scheff et al., 1997; Hunt et al., 2009, 2010, 2011, 2012). Briefly, animals were anesthetized using 2% isoflurane and placed in a stereotaxic frame. A midline incision was made to reveal the skull and a 5 mm craniotomy was performed lateral to the sagittal suture and centered between bregma and lambda. The skull cap was removed without damage to the underlying dura. A computer controlled, pneumatically driven impactor fitted with a 3 mm stainless-steel beveled tip (TBI-0310; Precision Systems and Instrumentation, Fairfax Station, VA, United States) was used to compress the cortex without breaking the dura mater. Impact parameters were set to 1.0 mm depth (hard stop), 3.5 m/s velocity, and 500 ms duration. For sham-injured controls, craniotomy was performed but no impact was administered to the brain. The incision was sutured and the animals were allowed to recover. All mice given CCI survived and remained otherwise healthy until experimentation. These parameters result in a well-characterized injury that predisposes mice to development of posttraumatic epilepsy by 8–12 weeks post-injury (Hunt et al., 2009, 2010, 2011, 2012).

### Hippocampal Slice Preparation

Mice were anesthetized by isoflurane inhalation to effect (lack of tail pinch response) and decapitated while anesthetized. The brain was then rapidly removed and immediately immersed in ice-cold (0–4°C), oxygenated (95% O_2_–5% CO_2_) artificial cerebrospinal fluid (ACSF) containing (in mM) 124 NaCl, 3 KCl, 26 NaHCO_3_, 1.4 NaH_2_PO_4_, 11 glucose, 1.3 CaCl_2_, 1.3 MgCl_2_; 1.0 kynurenic acid (KYN; Sigma-Aldrich, St. Louis, MO, United States); pH = 7.2–7.4, with an osmolality of 290–305 mOsmol/kg H_2_O. The brain was blocked, mounted on a sectioning stage, and 350 μm slices were cut in the coronal plane with a vibrating microtome (Vibratome Series 1000; Technical Products International, St. Louis, MO, United States) to ensure consistency of transverse slices from the dorsal one-third of the hippocampus. Each hippocampus was isolated from surrounding brain areas, making sure to completely remove entorhinal cortex, and slice order was maintained so that the location relative to the injury within each hippocampus was known. The slices were then transferred to a holding chamber where they were superfused with warmed (32–34°C) ACSF.

### Electrophysiology

After an equilibration period of at least 1 h, slices were transferred to a recording chamber on an upright, fixed-stage microscope equipped with infrared, differential interference contrast optics (IR-DIC; Olympus BX51WI, Melville, NY, United States), where they were continually superfused with warmed (32–34°C) ACSF. The ACSF always contained the glutamate receptor antagonist KYN (1 mM) and was identical to that used in the dissection and recovery, except when drugs [e.g., bicuculline methiodide (BIC) or zolpidem] were added, as described. Whole-cell patch-clamp recordings were performed from visualized DGCs. Recording pipettes were pulled (P-87, Sutter Instruments; Novato, CA, United States) from borosilicate glass capillaries with 1.65 mm outer diameter and 0.45 mm wall thickness (King Precision Glass, Claremont, CA, United States). Open tip resistance was 2–5 MΩ, seal resistance was 1–5 GΩ, series resistance was ≤20 MΩ (mean = 8.89 ± 0.60 MΩ, *n* = 79), uncompensated. Recordings were discontinued and not analyzed if series resistance varied by more than 25% during the recording. Recording pipettes were filled with (in mM) 140 Cs-gluconate, 1 NaCl, 5 EGTA, 10 HEPES, 1 MgCl_2_, 1 CaCl_2_, 3 CsOH, 2 ATP; pH = 7.15–7.30. A minimum of 5 min following establishment of whole-cell configuration was used in order to allow equilibration of the intracellular and recording pipette contents. Neural activity was recorded using Axon Instruments Axopatch 200B and MultiClamp 700B patch-clamp amplifiers (Molecular Devices, Sunnyvale, CA, United States), acquired at 10–20 kHz and low-pass filtered at 5 kHz using Digidata 1320A and 1322A digitizers and pClamp 10.3 software (Molecular Devices). Synaptic currents were analyzed off-line on a PC-style computer with pCLAMP programs (Molecular Devices) or MiniAnalysis 6.0.3 (Synaptosoft, Decatur, GA, United States). A value of 3× the root mean squared noise level for a given recording was used as the detection limit for synaptic currents. Spontaneous inhibitory postsynaptic currents (sIPSCs) and tonic GABA_A_ currents (I_TonicGABA_) were recorded in voltage-clamp mode with a voltage command of 0 mV. Added to the ACSF for specific experiments were the following: zolpidem (*N*,*N*,6-Trimethyl-2-(4-methylphenyl)imidazo[1,2-a]pyridine-3-acetamide; 1 μM; Tocris Bioscience, Minneapolis, MN, United States) and BIC (30 μM; Tocris Bioscience, Minneapolis, MN, United States). Zolpidem and BIC were dissolved in ACSF. We did not test DGC responsiveness to the compound flumazenil, and/or its possible blocking effect on any observed zolpidem responses, due to the temporal constrains of the long-term whole-cell patch clamp recordings used in this study as well as possible changes in flumazenil-responsiveness following epileptogenic insults (Leroy et al., 2004). Unless otherwise stated, neurons were recorded for 5 min under baseline conditions, 10 min during zolpidem application, and then for 5 min during application of BIC. Tonic GABA_A_R currents were measured as the mean difference in holding current (during baseline or zolpidem) relative to BIC. Charge transfer for tonic and synaptic GABA_A_R currents was sampled by integrating the amplitude of these electrical signals (i.e., converting to Amperes/second (i.e., picoCoulombs, pC) during a standardized 60 s bin (i.e., units of pC/60). Synaptic charge transfer was sampled by determining the charge transfer of the mean sIPSC during this 60 s bin (to estimate Amperes/second) and then multiplying this value by the total number of sIPSC events that occurred within it. Tonic charge transfer was sampled as the mean tonic GABA_A_R current amplitude that occurred during this standardized 60 s bin (to estimate Amperes/second) and then multiplied by the bin duration (60 s). The ratio of charge transfer was calculated as (tonic GABA_A_R/synaptic GABA_A_R) to create values that were positive integers.

### RNA Isolation and cDNA

Isolated dentate gyrus (DG) was harvested from each hemisphere and processed according to previously described methodology (Hideo et al., 2009; Boychuk et al., 2016). The tissue was visualized with a dissecting microscope (SMZ800; Nikon, Melville, NY, United States) and was submerged in ice-cold (0–4°C) ACSF containing kynurenic acid for all steps of dissection. The dorsal half of each isolated DG was selected for transcript analysis because the greatest proportion of cell signaling changes in the ipsilateral DG (e.g., mossy fiber sprouting and interneuron loss) are observed in this region after CCI (Hunt et al., 2009, 2010, 2011, 2012). Resulting isolated, dorsal halves of DG ipsilateral and contralateral to CCI or sham-injury were treated as individual samples. Each sample was immediately homogenized in ice-cold 500 μL of TRIzol (Sigma-Aldrich). Chloroform (100–250 μL) was added and tubes were vortexed for 15 s and then maintained at 4°C for 20 min and subsequently centrifuged at 12,000 rpm for 15 min at 4°C. The pellet was discarded and the RNA supernatant was transferred into fresh 1.5 mL centrifuge tubes, mixed with 500 μL of ice-cold propanol, incubated at room temperature for 10 min, and centrifuged at 12,000 rpm for 10 min at 4°C. Propanol was decanted and RNA was washed by re-suspension in 500 μL 75% ethanol followed by centrifugation at 7500–12,000 rpm for 10 min at 4°C. The wash step was repeated, the ethanol decanted, and RNA samples were air-dried for 10–20 min. RNA was then re-dissolved in 20 μL ddH20. Spectrophotometry (NanoDrop, Fisher Scientific, Wilmington, DE, United States) was used to determine mRNA concentration and purity.

### TaqMan Quantitative Real-Time Polymerase Chain Reaction

Quantitative real-time polymerase chain reaction (qRT-PCR) was performed as previously described (Boychuk et al., 2016). Samples of mRNA (2 μg) were used to create cDNA using SuperScript II reverse transcriptase kit (Invitrogen, Carlsbad, CA, United States) using a Mastercycler (Eppendorf, Hauppauge, NY, United States). All qRT-PCR reactions were run in triplicate in 96-well optical grade plates using a 7500 Fast Real-Time PCR System (Applied Biosystems, Foster City, CA, United States). Each run consisted of 1 cycle of 50°C for 2 min, then 1 cycle of 95°C for 10 min, and 40 cycles of 95°C for 15 s, and 60°C for 1 min. Total volume for each run was 20 μL containing 10 μL of TaqMan 2× PCR Master Mix (Applied Biosystems), 1 μL of primer (Applied Biosystems) and a combination of cDNA and ddH_2_O that provided a cDNA concentration of 50 ng. All reactions used forward and reverse primer concentrations of 100 nM. Probe concentrations for all reactions were 50 nM. Primer and TaqMan probe sets were purchased from Applied Biosystems. The sequences for each were generated from the listed references within GenBank: α1: Mm00439046_m1; α2: Mm00433435_m1; α3: Mm01294271_m1; γ2: Mm00433489_m1; and β-actin: Mm00607939_s1. No-template and no-RT controls were run for each plate and results discarded if false positives were present. Delta cycle thresholds were used for statistical analysis (two-tail *T*-tests) of gene expression of GABA_A_R subunits and these data are presented as relative change using the 2^–ΔΔCT^ method (Wood and Giroux, 2003).

### Statistics

Repeated Measures 2-Way Analysis of Variance (RM 2-Way ANOVA) was used to test differences for all electrophysiology data followed by protected *post hoc* Tukey’s Multiple Comparisons to test between group differences and protected *post hoc* Sidak’s Multiple Comparisons to test within-group differences pre/post zolpidem. The outcome of main effects determined whether *post hoc* testing was performed (Wei et al., 2012). Percent relative change was calculated using the formula: [C = 100 × (Post − Pre/Pre)].

## Results

**FIGURE 1:**
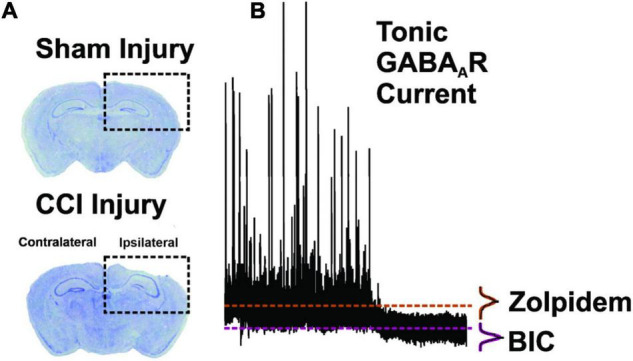
Methodology for brain injury and tonic GABA_A_R measurement. **(A)** Coronal brain sections of dorsal hippocampus stained with cresyl violet. In comparison to sham-injury (top panel), CCI-injury (bottom panel; 1.0 mm depth) resulted in cortical cavitation and distortion while sparing portions of the dentate gyrus (Hunt et al., 2009, 2010, 2012; Butler et al., 2015). **(B)** Representative neurophysiological recording from a DGC in order to depict measurement of the Tonic GABA_A_R at baseline and in the presence of zolpidem (1 μM) and the GABA_A_R antagonist, bicuculline methiodide (BIC; 30 μM). Mean current values during BIC application are subtracted from mean current values during zolpidem application to quantify the zolpidem-selective tonic GABA_A_R current (dashed lines). All points histograms, and subsequent Gaussian fits, are provided for each section of trace to demonstrate the population data from which mean values are derived.

Synaptic and tonic GABA_A_R-mediated currents were assessed in DGCs at 1–2 and 8–13 weeks following CCI injury (Figure 1). Recordings were made from DGCs ipsilateral to CCI injury (i.e., Ipsi-DGCs) and contralateral to injury (i.e., Contra-DGCs) or after sham injury (i.e., Sham-DGCs). Sample sizes for 1–2 weeks post-surgery were the following: Sham-DGCs, *n* = 13 cells (8 mice); Contra-DGCs, *n* = 13 cells (9 mice); Ipsi-DGCs, *n* = 14 cells (9 mice). Sample sizes for 8–13 weeks post-surgery were the following: Sham-DGCs, *n* = 10 cells (8 mice); Contra-DGCs, *n* = 15 cells (8 mice); Ipsi-DGCs, *n* = 15 cells (9 mice); the one exception is that sample size for tonic GABA_A_R current for Ipsi-DGCs was 14 (rather than 15) as one recording lacked sufficient duration of bicuculline application. A total of 1–3 cells were recorded per mouse and we assessed the potential that the recordings from any given mouse may have unduly biased the data (i.e., “nesting effect”) and found no such issues.

### Tonic GABA Receptor-Mediated Current 1–2 Weeks Post-injury

I_TonicGABA_ was evaluated in DGCs (Figure 1) because we have previously observed its sparing (and/or compensatory remodeling) in this cell type after CCI, findings that have recently been replicated (Boychuk et al., 2016; Butler et al., 2016; Becerra et al., 2021). This sparing of the baseline I_TonicGABA_ in Ipsi-DGCs is surprising because it occurs coincident with robust alterations to phasic GABA_A_R (I_SynapticGABA_) signaling as attributed to loss of select groups of GABA-expressing cells within the hippocampus (Pavlov et al., 2011). Further, we have previously found that Ipsi-DGCs exhibit an evoked I_TonicGABA_ that is less responsive to THIP, a GABA_A_R superagonist that predominantly targets GABA_A_Rs containing δ subunits along with α4 and α6 subunits (Saarelainen et al., 2008; Chandra et al., 2010; Boychuk et al., 2016; Butler et al., 2016; Becerra et al., 2021). Few, if any, reports exist for the effects of zolpidem on I_TonicGABA_ and I_SynapticGABA_ in DGCs after brain injury and the use of charge transfer provides helpful information regarding their relative contributions to GABA_A_R signaling (Bai et al., 2001).

**FIGURE 2:**
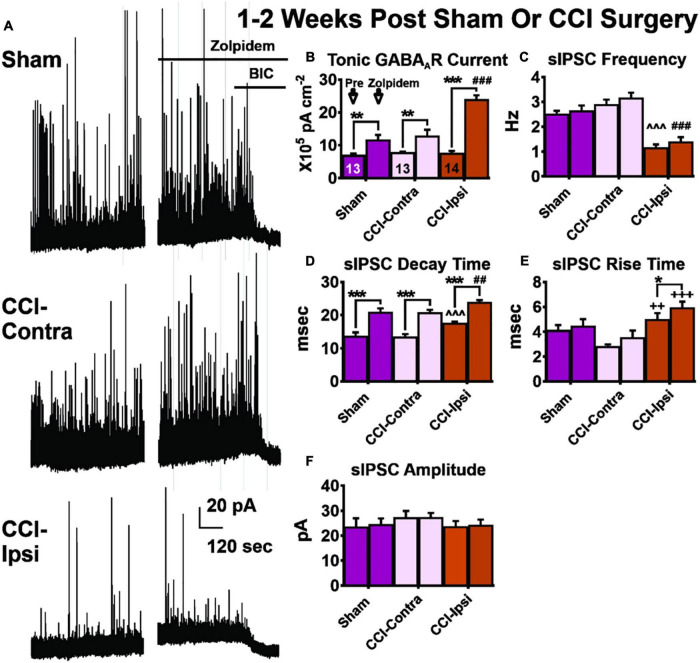
GABA receptor signaling in DGCs 1–2 weeks post-CCI before and after zolpidem application. Comparisons were made between DGCs from sham animals (i.e., Sham; *n* = 13 cells), DGCs contralateral to CCI injury (i.e., CCI-Contra; *n* = 13 cells), and DGCs ipsilateral to CCI injury (i.e., CCI-Ipsi; *n* = 14 cells). **(A)** Example traces showing the effect of zolpidem and bicuculline in DGCs recorded 1–2 weeks after CCI injury. **(B)** Baseline tonic GABA_A_R current is spared in CCI-Ipsi DGCs, whereas this current is profoundly augmented by zolpidem (85% increase vs. controls) in these cells after brain injury. **(C–E)** CCI-Ipsi DGCs also exhibit decreased sIPSC frequency and increased sIPSC decay time and rise time. Zolpidem augmented sIPSC decay time in all groups. **(F)** sIPSC amplitude was unaffected by zolpidem in any group at 1–2 weeks post-CCI injury. The symbol “*” denotes a significant within-group change between pre/post zolpidem. The symbol “^” denotes a significant change between the pre-zolpidem CCI-Ipsi group compared to both pre-zolpidem Sham- and CCI-Contra groups. The symbol “#” denotes a significant change between the post-zolpidem CCI-Ipsi group compared to both post-zolpidem Sham- and CCI-Contra groups. The symbol “+” denotes a significant difference between the CCI-Ipsi group compared to the CCI-Contra group (indicated as pre-zolpidem or post-zolpidem, as appropriate). Significance set as *p* ≤ 0.05; standard statistical codes were used to indicate level of significance (e.g., *p ≤ 0.05, **p ≤ 0.01, ***p ≤ 0.001).

For amplitude of baseline and zolpidem evoked I_TonicGABA_ (Figures 2A,B and Table 1), a significant main effect of group was detected [2-Way RM ANOVA; *F*(2,37) = 14.37, *p* < 0.0001]. Baseline I_TonicGABA_ amplitude of Ipsi-DGCs was comparable to Contra-DGCs (Tukey, *p* > 0.99) and Sham-DGCs (Tukey, *p* = 0.88). The zolpidem evoked I_TonicGABA_ in Ipsi-DGCs (Figures 2A,B and Table 1), was significantly greater than in Contra-DGCs (Tukey, *p* < 0.0001), and Sham-DGCs (Tukey, *p* < 0.0001). Comparison of the amplitude of zolpidem evoked I_TonicGABA_ between Sham- and Contra-DGCs revealed no significant differences before zolpidem (Tukey, *p* = 0.91) or after zolpidem (Tukey, *p* = 0.69). A significant main effect of zolpidem was also detected [2-Way RM ANOVA; *F*(1,37) = 78.99, *p* < 0.0001]. Zolpidem application evoked a change in the holding current in DGCs from all experimental groups thereby indicating a responsiveness of DGCs from all experimental groups to this compound. Amplitude of I_TonicGABA_ was increased by zolpidem treatment in Ipsi-DGCs (211% change; Sidak, *p* < 0.0001), Contra-DGCs (72% change; Sidak, *p* = 0.010), and in Sham-DGCs (68% change; Sidak, *p* = 0.031). A significant interaction between main effects was also detected [2-Way RM ANOVA; *F*(2,37) = 14.64, *p* < 0.0001].

**TABLE 1:** I_Tonic_ amplitude for dentate granule cells (DGCs) from Sham controls, contralateral (Contra), or ipsilateral (Ipsi) to CCI injury.

I_TonicGABA_ amplitude 1–2 weeks post-injury					

Baseline I_TonicGABA_ currents 1–2 weeks post-injury			Zolpidem-evoked I_TonicGABA_ currents 1–2 weeks post-injury		
	
Sham-DGCs	Contra-DGCs	Ipsi-DGCs	Sham-DGCs	Contra-DGCs	Ipsi-DGCs
6.77 ± 0.69 pA	7.51 ± 0.56 pA	7.61 ± 0.63 pA	11.40 ± 1.75 pA *	12.89 ± 1.79 pA *	23.69 ± 1.48 pA *^,#^

**I_TonicGABA_ amplitude 8–13 weeks post-injury**					

**Baseline I_TonicGABA_ currents 8–13 weeks post-injury**			**Zolpidem-evoked I_TonicGABA_ currents 8–13 weeks post-injury**		
	
**Sham-DGCs**	**Contra-DGCs**	**Ipsi-DGCs**	**Sham-DGCs**	**Contra-DGCs**	**Ipsi-DGCs**

6.83 ± 1.09 pA	7.5 ± 1.70 pA	10.52 ± 1.80 pA	8.74 ± 1.52 pA	8.59 ± 1.74 pA	15.26 ± 2.25 pA *^,#^

Mean ± SEM tonic current amplitude values are presented for baseline and zolpidem-evoked currents. The symbol “*” denotes a significant within-group change between pre/post zolpidem. The symbol “#” denotes a significant change between the post-zolpidem CCI-Ipsi group compared to both post-zolpidem Sham- and CCI-Contra groups.

### Spontaneous Inhibitory Postsynaptic Current Frequency 1–2 Weeks Post-injury

Reduced sIPSC frequency in Ipsi-DGCs after CCI injury (Figure 2C and Table 2) has been previously reported (Hunt et al., 2011; Boychuk et al., 2016; Butler et al., 2016; Zhu et al., 2019; Becerra et al., 2021), presumably due to selective loss of hippocampal GABA-expressing neurons (Lowenstein et al., 1992; Zhu et al., 1997; Pavlov et al., 2011; Butler et al., 2016, 2017; Frankowski et al., 2019). Zolpidem application in a mouse model of epilepsy was less effective in augmenting GABA potency in isolated DGCs (Brooks-Kayal et al., 1998), but it is unclear if zolpidem application augments spontaneous inhibitory inputs onto DGCs in slices after brain injury. For sIPSC frequency, a significant main effect of group was detected [2-Way RM ANOVA; *F*(2,37) = 24.64, *p* < 0.0001]. Baseline sIPSC frequency (Figure 2C and Table 2) was significantly lower in Ipsi-DGCs in comparison to Contra-DGCs (Tukey, *p* < 0.0001) and Sham-DGCs (Tukey, *p* < 0.0001), similar to previous reports (Hunt et al., 2011; Boychuk et al., 2016; Butler et al., 2016; Becerra et al., 2021). A significant main effect of zolpidem was also detected [2-Way RM ANOVA; *F*(1,37) = 13.2, *p* = 0.0008]. In the presence of zolpidem, sIPSC frequency in Ipsi-DGCs remained reduced relative to the other groups of Contra-DGCs (Tukey, *p* < 0.0001 vs. Ipsi-DGCs), and Sham-DGCs (Tukey, *p* < 0.0001 vs. Ipsi-DGCs). No significant differences in sIPSC frequency were observed between Sham- and Contra-DGCs before (Tukey, *p* = 0.32) or after zolpidem (Tukey, *p* = 0.18). A significant main effect of and zolpidem was also detected [2-Way RM ANOVA; *F*(1,37) = 13.2, *p* = 0.0008]. However, there were no significant within-group differences in sIPSC frequency with zolpidem treatment. Instead, during *post hoc* testing, statistical trends (i.e., *p* < 0.10) were observed for changes in sIPSC frequency due to zolpidem treatment in some cases: Ipsi-DGCs (21% change; Sidak, *p* = 0.082), Contra-DGCs (9% change; Sidak, *p* = 0.059) and Sham-DGCs (7% change; Sidak, *p* = 0.32). The interaction between main effects was non-significant [2-Way RM ANOVA; *F*(2,37) = 0.1939, *p* = 0.82].

**TABLE 2:** I_Synaptic_ frequency, kinetics, and amplitude properties 1–2 weeks post-injury.

I_Synaptic_ frequency 1–2 weeks post-injury					

Baseline sIPSC frequency 1–2 weeks post-injury			Post-zolpidem sIPSC frequency 1–2 weeks post-injury		
	
Sham-DGCs	Contra-DGCs	Ipsi-DGCs	Sham-DGCs	Contra-DGCs	Ipsi-DGCs
2.48 ± 0.17 Hz	2.87 ± 0.22 Hz	1.16 ± 0.12 Hz ^	2.65 ± 0.20 Hz	3.14 ± 0.24 Hz	1.40 ± 0.18 Hz #

**I_Synaptic_ rise time 1–2 weeks post-injury**					

**Pre-zolpidem sIPSC rise time 1–2 weeks post-injury**			**Post-zolpidem sIPSC rise time 1–2 weeks post-injury**		
	
**Sham-DGCs**	**Contra-DGCs**	**Ipsi-DGCs**	**Sham-DGCs**	**Contra-DGCs**	**Ipsi-DGCs**

4.01 ± 0.48 ms	2.84 ± 0.15 ms	5.01 ± 0.48 ms	4.47 ± 0.52 ms	3.48 ± 0.61 ms	5.96 ± 0.46 ms

**I_Synaptic_ decay constant 1–2 weeks post-injury**					

**Pre-zolpidem sIPSC decay time 1–2 weeks post-injury**			**Post-zolpidem sIPSC decay time 1–2 weeks post-injury**		
	
**Sham-DGCs**	**Contra-DGCs**	**Ipsi-DGCs**	**Sham-DGCs**	**Contra-DGCs**	**Ipsi-DGCs**

13.76 ± 1.01 ms	13.53 ± 0.71 ms	17.64 ± 0.41 ms ^	20.98 ± 1.04 ms *	20.83 ± 0.79 ms *	24.00 ± 0.51 ms *^,#^

**I_Synaptic_ amplitudes 1–2 weeks post-injury**					

**Pre-zolpidem sIPSC amplitude 1–2 weeks post-injury**			**Post-zolpidem sIPSC amplitude 1–2 weeks post-injury**		
	
**Sham-DGCs**	**Contra-DGCs**	**Ipsi-DGCs**	**Sham-DGCs**	**Contra-DGCs**	**Ipsi-DGCs**

23.63 ± 3.32 pA	26.95 ± 2.97 pA	23.79 ± 2.0 pA	24.61 ± 2.31 pA	27.00 ± 2.12 pA	24.33 ± 2.15 pA

*Mean ± SEM sIPSC frequency, rise time, decay constant, and amplitude values during baseline and zolpidem application are presented.*

The symbol “*” denotes a significant within-group change between pre/post zolpidem. The symbol “^” denotes a significant change between the pre-zolpidem CCI-Ipsi group compared to both pre-zolpidem Sham- and CCI-Contra groups. The symbol “#” denotes a significant change between the post-zolpidem CCI-Ipsi group compared to both post-zolpidem Sham- and CCI-Contra groups.

### Spontaneous Inhibitory Postsynaptic Current Decay Time 1–2 Weeks Post-injury

Spontaneous inhibitory postsynaptic current decay time (Figure 2D and Table 2) was evaluated here because increased sIPSC decay times in Ipsi-DGCs have been reported previously (Hunt et al., 2011; Boychuk et al., 2016; Butler et al., 2016; Becerra et al., 2021), and zolpidem may increase sIPSC decay time by acting at the benzodiazepine binding site, predominantly through augmentation of α_1_ subunit-containing GABA_A_Rs (Hiu et al., 2016). For sIPSC decay time, a significant main effect of group was detected [2-Way RM ANOVA; *F*(2,37) = 8.14, *p* = 0.0012]. Baseline sIPSC decay time (Figure 2D and Table 2) was significantly greater in Ipsi-DGCs in comparison to Contra-DGCs (30% difference; Tukey, *p* = 0.0008) and Sham-DGCs (28% difference; Tukey, *p* = 0.0016). In the presence of zolpidem, sIPSC decay time was significantly greater in Ipsi-DGCs in comparison to Contra-DGCs (15% change; Tukey, *p* = 0.012) and Sham-DGCs (14% change; Tukey, *p* = 0.018). The comparison of sIPSC decay time between Sham- and Contra-DGCs revealed no significant differences before (Tukey, *p* = 0.98) or after zolpidem (Tukey, *p* = 0.99). A significant main effect of zolpidem was detected [2-Way RM ANOVA; *F*(1,37) = 589.9, *p* < 0.0001]. Importantly, sIPSC decay time was increased between baseline and zolpidem treatment in Ipsi-DGCs (36% change; Sidak, *p* < 0.0001), Contra-DGCs (54% change; Sidak, *p* < 0.0001) and Sham-DGCs (10% change; Sidak, *p* < 0.0001). However, The interaction between main effects was non-significant [2-Way RM ANOVA; *F*(2,37) = 1.14, *p* = 0.33].

### Spontaneous Inhibitory Postsynaptic Current Rise Time 1–2 Weeks Post-injury

Increased sIPSC rise times (Figure 2E and Table 2) in Ipsi-DGCs have been reported previously and is thought to be related to altered functional GABA_A_R’s expressed by Ipsi-DGCs after CCI injury (Brooks-Kayal et al., 1998, 2001; Boychuk et al., 2016; Butler et al., 2016; Becerra et al., 2021). For sIPSC rise time, a significant main effect of effect of group was detected [2-Way RM ANOVA; *F*(2,37) = 7.33, *p* = 0.0021]. Baseline sIPSC rise time (Figure 2E and Table 2) was significantly greater in Ipsi-DGCs compared to Contra-DGCs (76% greater; Tukey, *p* = 0.0045) whereas this effect was not detected relative to Sham-DGCs (24% greater; Tukey, *p* = 0.32). In the presence of zolpidem, sIPSC rise time remained significantly greater in Ipsi-DGCs in comparison to Contra-DGCs (71% greater; Tukey, *p* = 0.0010) whereas only a statistical trend (i.e., *p* < 0.10) was observed between Ipsi-DGCs relative to Sham-DGCs (33%; Tukey, *p* = 0.071). The comparison of sIPSC rise time between Sham- and Contra-DGCs revealed no significant differences before zolpidem (Tukey, *p* = 0.18) or after zolpidem (Tukey, *p* = 0.31). A significant main effect of zolpidem was also detected [2-Way RM ANOVA; *F*(1,37) = 9.51, *p* = 0.0038]. Interestingly, sIPSC rise time was only increased between baseline and zolpidem treatment in Ipsi-DGCs (19% change; Sidak, *p* = 0.041) whereas there was no significant effect in Contra-DGCs (23% change; Sidak, *p* = 0.27) and Sham-DGCs (10% change; Sidak, *p* = 0.62). The interaction between main effects was non-significant [2-Way RM ANOVA; *F*(2,37) = 0.50, *p* = 0.61]. Overall, this suggests that zolpidem application in the slice circuit was only partially capable of augmenting the activation of synaptic GABA_A_Rs of DGCs.

### Spontaneous Inhibitory Postsynaptic Current Amplitude 1–2 Weeks Post-injury

For sIPSC amplitude (Figure 2F and Table 2), there were no significant main effects for group [2-Way RM ANOVA; *F*(2,37) = 0.48, *p* = 0.62], zolpidem [2-Way RM ANOVA; *F*(1,37) = 0.34, *p* = 0.56], or the interaction between main effects [2-Way RM ANOVA; *F*(2,37) = 0.090, *p* = 0.91]. sIPSC amplitudes at baseline, and in the presence of zolpidem, are provided in Table 2 and are similar to previous reports (Brooks-Kayal et al., 1998, 2001; Boychuk et al., 2016; Butler et al., 2016; Becerra et al., 2021). Interestingly, zolpidem failed to significantly increase sIPSC amplitude under these experimental conditions; similar findings have been noted in other cortical cell types (Hiu et al., 2016).

### Tonic GABA Receptor-Mediated Currents 8–13 Weeks Post-injury

**FIGURE 3:**
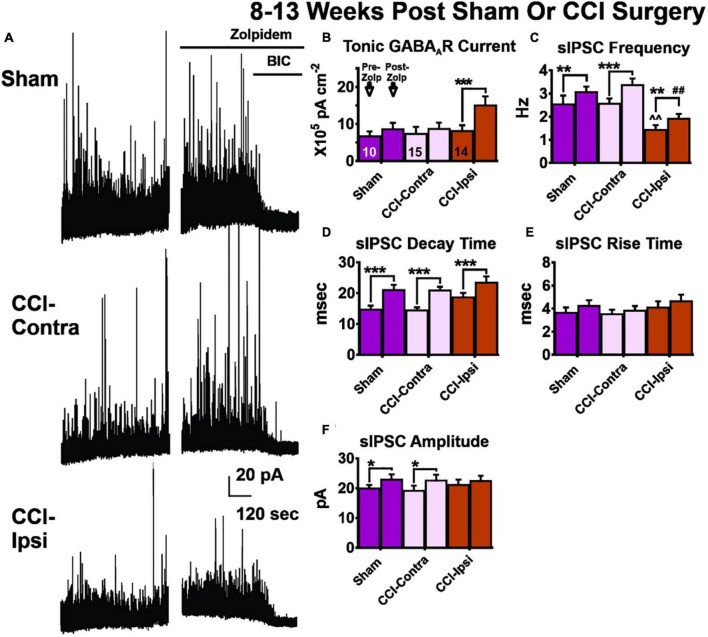
GABA receptor signaling in DGCs 8–13 weeks post-CCI before and after zolpidem application. Comparisons were made between DGCs from sham animals (i.e., Sham; *n* = 10 cells), DGCs contralateral to CCI injury (i.e., CCI-Contra; *n* = 14 cells for tonic GABA_A_R current; *n* = 15 cells for sIPSC parameters) and DGCs ipsilateral to CCI injury (i.e., CCI-Ipsi; *n* = 14 cells). **(A)** Example traces showing the effect of zolpidem and bicuculline in DGCs recorded 8–13 weeks after CCI injury. **(B)** Baseline tonic GABA_A_R current is spared in CCI-Ipsi DGCs whereas in the presence of zolpidem this current is 75% larger than in Sham-DGCs and 78% larger than in Contra-DGCs. **(C–E)** CCI-Ipsi DGCs also exhibit decreased sIPSC frequency and increased sIPSC decay time and rise time. Zolpidem augmented sIPSC decay time in all groups. **(F)** Zolpidem effect on sIPSC amplitude. The change in sIPSC amplitudes from baseline to zolpidem application was significantly increased in Sham-DGCs (15% change; Sidak, *p* = 0.035) and was significantly increased in Contra-DGCs (18% change; Sidak, *p* = 0.035) whereas no significant change was detected in Ipsi-DGCs (6% change; Sidak, *p* = 0.40). The symbol “*” denotes a significant within-group change between pre/post zolpidem. The symbol “^” denotes a significant change between the pre-zolpidem CCI-Ipsi group compared to both pre-zolpidem Sham and CCI-Contra groups. The symbol “#” denotes a significant change between the post-zolpidem CCI-Ipsi group compared to both post-zolpidem Sham and CCI-Contra groups. Significance set as *p* ≤ 0.05; standard statistical codes were used to indicate level of significance (e.g., *p ≤ 0.05, **p ≤ 0.01, ***p ≤ 0.001).

*De novo* development of excitatory synaptic inputs onto surviving GABAergic interneurons after CCI injury may be a compensatory mechanism for loss of synaptic inhibition onto Ipsi-DGCs (Hunt et al., 2011; Boychuk et al., 2016; Butler et al., 2016, 2017; Becerra et al., 2021), but we and others have also reported previously that I_TonicGABA_ is unaltered several weeks after CCI injury (Boychuk et al., 2016; Butler et al., 2016, 2017; Becerra et al., 2021). For amplitude of baseline I_TonicGABA_ (Figure 3A and Table 1), there was no significant main effect of group [2-Way RM ANOVA; *F*(2,36) = 2.06, *p* = 0.14]. A significant main effect of zolpidem was detected for amplitude of I_TonicGABA_ [2-Way RM ANOVA; *F*(1,36) = 14.59, *p* = 0.0005]. The application of zolpidem evoked a change in the holding current (Figures 3A,B and Table 1) in Ipsi-DGCs (45% change; Sidak, *p* < 0.0001), whereas this effect was not detected in Contra-DGCs (15% change; Sidak, *p* = 0.82) and Sham-DGCs (28% change; Sidak, *p* = 0.61), demonstrating that zolpidem application augments tonic inhibition prevalently in Ipsi-DGCs following CCI injury. The interaction between main effects was significant [2-Way RM ANOVA; *F*(2,36) = 4.90, *p* = 0.013].

### Spontaneous Inhibitory Postsynaptic Current Frequency 8–13 Weeks Post-injury

We previously reported that sIPSC frequency in Ipsi-DGCs remains reduced 8–13 weeks after injury, despite *de novo* development of excitatory synaptic inputs onto surviving GABAergic interneurons following CCI injury (Hunt et al., 2011; Boychuk et al., 2016; Butler et al., 2016, 2017). It is possible zolpidem could augment inhibitory input and we tested this possibility similarly to our measurements 1–2 weeks after CCI injury. For sIPSC frequency (Figure 3C and Table 3), a significant main effect of group was detected [2-Way RM ANOVA; *F*(2,37) = 9.72, *p* = 0.0004]. Baseline sIPSC frequency was significantly reduced in Ipsi-DGCs (Figure 3C and Table 3) compared to Contra-DGCs (43% change; Tukey, *p* = 0.0032) and Sham-DGCs (43% change; Tukey, *p* = 0.0078), similar to previous reports (Hunt et al., 2011; Boychuk et al., 2016; Butler et al., 2016; Zhu et al., 2019; Becerra et al., 2021). sIPSC frequency was still significantly reduced following zolpidem application in DGCs ipsilateral to CCI compared to DGCs contralateral to CCI (42% change; Tukey, *p* < 0.0001) and DGCs from Sham animals (37% change; Tukey, *p* = 0.0054). No differences in sIPSC frequency between Sham- and Contra-DGCs were observed before (Tukey, *p* > 0.99) or after zolpidem (Tukey, *p* = 0.74). A significant main effect of zolpidem was also detected [2-Way RM ANOVA; *F*(1,37) = 52.68, *p* < 0.0001]. Zolpidem treatment significantly increased sIPSC frequency in all three groups (Figure 3C and Table 3): Ipsi-DGCs (33% change; Sidak, *p* = 0.0029), Contra-DGCs (32% change; Sidak, *p* < 0.0001) and Sham-DGCs (20% change; Sidak, *p* = 0.0084) all exhibited significant increases in sIPSC frequency by zolpidem. The interaction between main effects was non-significant [2-Way RM ANOVA; *F*(2,37) = 1.75, *p* = 0.19]. These results demonstrate that zolpidem application equivalently impacts sIPSC frequency across experimental groups.

**TABLE 3:** I_Synaptic_ frequency, kinetics, and amplitude properties 8–13 weeks post-injury.

I_Synaptic_ frequency 8–13 weeks post-injury					

Baseline sIPSC frequency 8–13 weeks post-injury			Post-zolpidem sIPSC frequency 8–13 weeks post-injury		
	
Sham-DGCs	Contra-DGCs	Ipsi-DGCs	Sham-DGCs	Contra-DGCs	Ipsi-DGCs
2.57 ± 0.35 Hz	2.55 ± 0.24 Hz	1.46 ± 0.18 Hz ^	3.094 ± 0.21 Hz *	3.36 ± 0.29 Hz *	1.94 ± 0.18 Hz *^,#^

**I_Synaptic_ rise time 8–13 weeks post-injury**					

**Pre-zolpidem sIPSC rise time 8–13 weeks post-injury**			**Post-zolpidem sIPSC rise time 8–13 weeks post-injury**		
	
**Sham-DGCs**	**Contra-DGCs**	**Ipsi-DGCs**	**Sham-DGCs**	**Contra-DGCs**	**Ipsi-DGCs**

3.70 ± 0.40 ms	3.58 ± 0.31 ms	4.16 ± 0.48 ms	4.29 ± 0.43 ms	3.88 ± 0.34 ms	4.70 ± 0.51 ms

**I_Synaptic_ decay constant 8–13 weeks post-injury**					

**Pre-zolpidem sIPSC decay time 8–13 weeks post-injury**			**Post-zolpidem sIPSC decay time 8–13 weeks post-injury**		
	
**Sham-DGCs**	**Contra-DGCs**	**Ipsi-DGCs**	**Sham-DGCs**	**Contra-DGCs**	**Ipsi-DGCs**

14.94 ± 0.98 ms	14.66 ± 0.70 ms	18.92 ± 1.18 ms	21.26 ± 1.43 ms *	21.10 ± 0.94 ms *	23.69 ± 1.69 ms *

**I_Synaptic_ amplitudes 8–13 weeks post-injury**					

**Pre-zolpidem sIPSC amplitude 8–13 weeks post-injury**			**Post-zolpidem sIPSC amplitude 8–13 weeks post-injury**		
	
**Sham-DGCs**	**Contra-DGCs**	**Ipsi-DGCs**	**Sham-DGCs**	**Contra-DGCs**	**Ipsi-DGCs**

20.15 ± 0.95 pA	19.44 ± 1.38 pA	21.35 ± 1.50 pA	23.11 ± 1.50 pA	22.85 ± 1.70 pA	22.67 ± 1.44 pA

Mean ± SEM sIPSC frequency, rise time, decay constant, and amplitude values during baseline and zolpidem application are presented. The symbol “*” denotes a significant within-group change between pre/post zolpidem. The symbol “^” denotes a significant change between the pre-zolpidem CCI-Ipsi group compared to both pre-zolpidem Sham- and CCI-Contra groups. The symbol “#” denotes a significant change between the post-zolpidem CCI-Ipsi group compared to both post-zolpidem Sham- and CCI-Contra groups.

### Spontaneous Inhibitory Postsynaptic Current Decay Time 8–13 Weeks Post-injury

For sIPSC decay time (Figure 3D and Table 3), only a statistical trend (i.e., *p* < 0.10) was observed for the overall effect of group [2-Way RM ANOVA; *F*(2,37) = 3.04, *p* = 0.060]. An overall effect of zolpidem was detected [2-Way RM ANOVA; *F*(1,37) = 143.8, *p* < 0.0001], wherein sIPSC decay time of Ipsi-DGCs (25% change; Sidak, *p* < 0.0001), Contra-DGCs (44% change; Sidak, *p* < 0.0001), and Sham-DGCs (42% change; Sidak, *p* < 0.0001) were significantly augmented by zolpidem in all groups. The interaction between main effects was non-significant [2-Way RM ANOVA; *F*(2,37) = 1.36, *p* = 0.27].

### Spontaneous Inhibitory Postsynaptic Current Rise Time 8–13 Weeks Post-injury

For sIPSC rise time (Figure 3E and Table 3), no overall effect was detected for group [2-Way RM ANOVA; *F*(2,37) = 0.97, *p* = 0.39]. An overall effect of zolpidem was detected [2-Way RM ANOVA; *F*(1,37) = 4.62, *p* = 0.038], however, no significant *post hoc* relationships were detected for zolpidem during multiple comparison testing: Ipsi-DGCs (13% change; Sidak, *p* = 0.36), Contra-DGCs (8% change; Sidak, *p* = 0.80), or Sham-DGCs animals (16% change; Sidak, *p* = 0.45). The interaction between main effects was non-significant [2-Way RM ANOVA; *F*(2,37) = 0.18, *p* = 0.83].

### Spontaneous Inhibitory Postsynaptic Current Amplitude 8–13 Weeks Post-injury

For sIPSC amplitude (Figure 3F and Table 3), no main effect was detected for group [2-Way RM ANOVA; *F*(2,37) = 0.11, *p* = 0.90]. An overall main effect was detected for zolpidem treatment on sIPSC amplitude [2-Way RM ANOVA; *F*(1,37) = 20.26, *p* < 0.001]. The change in sIPSC amplitudes, from baseline to zolpidem application (Figure 3E and Table 3), was significantly increased in Sham-DGCs (15% change; Sidak, *p* = 0.035) and Contra-DGCs (18% change; Sidak, *p* = 0.035) whereas no significant change was detected in Ipsi-DGCs (6% change; Sidak, *p* = 0.40). No significant interaction was detected between main effects [2-Way RM ANOVA; *F*(2,37) = 1.42, *p* = 0.25].

### Charge Transfer of Tonic GABA Receptor Current and Synaptic GABA Receptor Currents

Ipsi-DGCs exhibit several differences from uninjured control conditions (Sham and contralateral to injury) such as kinetic properties of GABA_A_R sIPSCs as well as a profound reduction in sIPSC frequency and increase in zolpidem augmentation of their tonic GABA_A_R current (as assessed by % change). To better understand the overall effect of these relationships we calculated charge transfer of these signaling modalities for I_SynapticGABA_ and I_TonicGABA_. Charge transfer is an “area under the curve”-type measurement of overall current over a matching duration of time that allows direct comparison of the contribution of tonic and synaptic GABA_A_R signaling and provides a separate way to analyze zolpidem augmentation of these signaling modalities since zolpidem affects many kinetic parameters simultaneously. Importantly, the ratio of charge transfer (I_TonicGABA_/I_SynapticGABA_) provides a means to test whether zolpidem augmentation predominantly occurs in tonic or phasic GABA_A_R signaling, as data here indicate that zolpidem profoundly augments I_TonicGABA_ in Ipsi-DGCs whereas zolpidem augmentation mainly impacts I_SynapticGABA_ in DGCs from uninjured control conditions.

### Tonic GABA Receptor Charge Transfer 1–2 Weeks Post-injury

**FIGURE 4:**
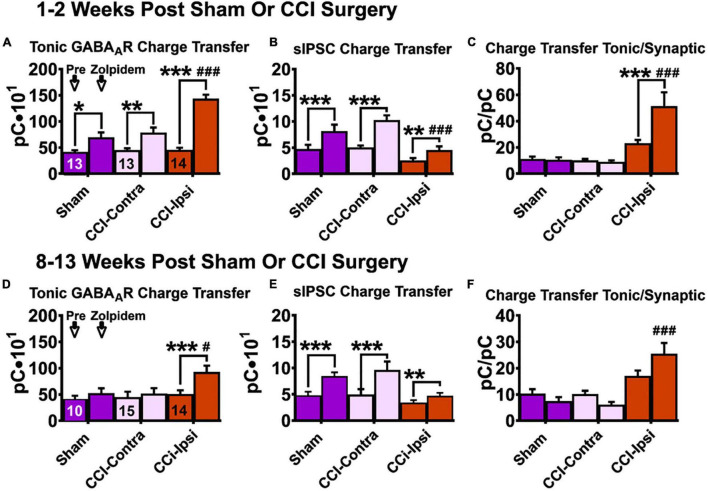
Charge transfer of tonic and synaptic GABA_A_R signaling in DGCs following CCI injury at baseline and after zolpidem application at 1–2 weeks and 8–13 weeks post-injury. Comparisons were made between DGCs from sham animals (i.e., Sham; *n* = 10 cells), DGCs contralateral to CCI injury (i.e., CCI-Contra; *n* = 15 cells) and DGCs ipsilateral to CCI injury (i.e., CCI-Ipsi; *n* = 14 cells). Tonic charge transfer is significantly augmented by zolpidem in DGCs from ipsilateral hemisphere of injured animals at 1–2 weeks **(A)** and 8–13 weeks **(D)** post-CCI. Synaptic charge transfer is significantly augmented by zolpidem in DGCs from all groups 1–2 weeks after injury **(B)** whereas this augmentation was less impactful in DGCs ipsilateral to CCI 8–13 weeks after injury **(E)**. Ratio of baseline charge transfer (tonic/synaptic) is elevated in DGCs ipsilateral to CCI at 1–2 weeks **(C)** and at 8–13 weeks **(F)** after injury, whereas in the presence of zolpidem this ratio is larger at both 1–2 weeks post injury (382% larger than in Sham-DGCs and 499% larger than in Contra-DGCs) and 8–13 weeks post-injury (251% larger than in Sham-DGCs and 315% larger than in Contra-DGCs). The symbol “*” denotes a significant within-group change between pre/post zolpidem. The symbol “#” denotes a significant change between the post-zolpidem CCI-Ipsi group compared to both post-zolpidem Sham and CCI-Contra groups. Significance set as *p* ≤ 0.05; standard statistical codes were used to indicate level of significance (e.g., *p ≤ 0.05, **p ≤ 0.01, ***p ≤ 0.001).

For tonic charge transfer at 1–2 weeks post-CCI (Figure 4A and Table 4), a main effect of group was detected [2-Way RM ANOVA; *F*(2,37) = 14.37, *p* < 0.0001]. At baseline conditions, Ipsi-DGCs exhibit tonic charge transfer that is not different than Contra-DGCs (Tukey, *p* > 0.99) or Sham-DGCs (Tukey, *p* = 0.88). Markedly, in the presence of zolpidem, Ipsi-DGCs exhibit tonic charge transfer that is significantly greater (84%) than Contra-DGCs (Tukey, *p* < 0.0001) and is significantly greater (108%) than Sham-DGCs (Tukey, *p* < 0.0001). There was no difference in tonic charge transfer between Sham- and Contra-DGCs before zolpidem (Tukey, *p* = 0.91) or after zolpidem (Tukey, *p* = 0.69). A main effect of zolpidem was detected [2-Way RM ANOVA; *F*(1,37) = 78.99, *p* < 0.0001]. Zolpidem-mediated I_TonicGABA_ charge transfer was greater in Ipsi-DGCs (211%; Sidak, *p* < 0.0001), Contra-DGCs (72%; Sidak, *p* = 0.010), and Sham-DGCs (68%; Sidak, *p* = 0.031) than their respective baseline values. A significant interaction between main effects was detected [2-Way RM ANOVA; *F*(2,37) = 14.64, *p* < 0.0001]. These results demonstrate a significant influence of zolpidem on inhibitory tone mediated by I_TonicGABA_ in Ipsi-DGCs at an early time following CCI injury.

**TABLE 4:** I_Tonic_, I_Synaptic_ charge transfer, and I_Tonic_/I_Synaptic_ charge transfer ratio.

I_Tonic_ charge transfer 1–2 weeks post-injury					

Baseline I_Tonic_ charge transfer 1–2 weeks post-injury			Post-zolpidem I_Tonic_ charge transfer 1–2 weeks post-injury		
	
						Sham-DGCs	Contra-DGCs	Ipsi-DGCs	Sham-DGCs	Contra-DGCs	Ipsi-DGCs
40.63 ± 4.16 pC10^1^	45.06 ± 3.36 pC10^1^	45.66 ± 3.79 pC10^1^	68.39 ± 10.47 pC10^1^ *	77.36 ± 10.76 pC10^1^ *	142.12 ± 8.90 pC10^1^ *^,#^

**I_Synaptic_ charge transfer 1–2 weeks post-injury**					

**Baseline I_Synaptic_ charge transfer 1–2 weeks post-injury**			**Post-zolpidem I_Synaptic_ charge transfer 1–2 weeks post-injury**		
	
**Sham-DGCs**	**Contra-DGCs**	**Ipsi-DGCs**	**Sham-DGCs**	**Contra-DGCs**	**Ipsi-DGCs**

4.61 ± 0.92 pC10^1^	4.91 ± 0.47 pC10^1^	2.55 ± 0.46 pC10^1^ ^	8.14 ± 1.24 pC10^1^ *	10.14 ± 1.03 pC10^1^ *	4.52 ± 0.72 pC10^1^ *^,#^

**I_Tonic_/I_Synaptic_ charge transfer ratio 1–2 weeks post-injury**					

**Baseline I_Tonic_/I_Synaptic_ charge transfer ratio 1–2 weeks post-injury**			**Post-zolpidem I_Tonic_/I_Synaptic_ charge transfer ratio 1–2 weeks post-injury**		
	
**Sham-DGCs**	**Contra-DGCs**	**Ipsi-DGCs**	**Sham-DGCs**	**Contra-DGCs**	**Ipsi-DGCs**

11.17 ± 1.77 a.u.	10.08 ± 1.12 a.u.	22.63 ± 3.00 a.u. ^	10.55 ± 1.86 a.u.	8.49 ± 1.52 a.u.	50.82 ± 11.05 a.u. *^,#^

**I_Tonic_ charge transfer 8–13 weeks post-injury**					

**Baseline I_Tonic_ charge transfer 8–13 weeks post-injury**			**Zolpidem-evoked I_Tonic_ charge transfer 8–13 weeks post-injury**		
	
**Sham-DGCs**	**Contra-DGCs**	**Ipsi-DGCs**	**Sham-DGCs**	**Contra-DGCs**	**Ipsi-DGCs**

41.01 ± 6.56 pC10^1^	45.02 ± 10.15 pC10^1^	49.61 ± 8.15 pC10^1^	52.44 ± 9.14 pC10^1^	51.55 ± 10.46 pC10^1^	91.35 ± 13.29 pC10^1^ *^,#^

**I_Synaptic_ charge transfer 8–13 weeks post-injury**					

**Baseline I_Synaptic_ charge transfer 8–13 weeks post-injury**			**Zolpidem-evoked I_Synaptic_ charge transfer 8–13 weeks post-injury**		
	
**Sham-DGCs**	**Contra-DGCs**	**Ipsi-DGCs**	**Sham-DGCs**	**Contra-DGCs**	**Ipsi-DGCs**

4.68 ± 0.80 pC10^1^	4.83 ± 1.15 pC10^1^	3.41 ± 0.47 pC10^1^	8.45 ± 0.71 pC10^1^ *	9.51 ± 1.72 pC10^1^ *	4.71 ± 0.57 pC10^1^^#^

**I_Tonic_/I_Synaptic_ charge transfer ratio 8–13 weeks post-injury**					

**Baseline I_Tonic_/I_Synaptic_ charge transfer ratio 8–13 weeks post-injury**			**Post-zolpidem I_Tonic_/I_Synaptic_ charge transfer ratio 8–13 weeks post-injury**		
	
**Sham-DGCs**	**Contra-DGCs**	**Ipsi-DGCs**	**Sham-DGCs**	**Contra-DGCs**	**Ipsi-DGCs**

10.26 ± 1.75 a.u.	10.12 ± 1.27 a.u.	16.80 ± 2.26 a.u.	7.17 ± 1.78 a.u.	6.08 ± 1.09 a.u.	25.19 ± 4.34 a.u. *^,#^

Mean ± SEM calculated charge transfer values and I_Tonic_/I_Synaptic_ ratios are presented. The symbol “*” denotes a significant within-group change between pre/post zolpidem. The symbol “^” denotes a significant change between the pre-zolpidem CCI-Ipsi group compared to both pre-zolpidem Sham- and CCI-Contra groups. The symbol “#” denotes a significant change between the post-zolpidem CCI-Ipsi group compared to both post-zolpidem Sham- and CCI-Contra groups.

### Synaptic GABA Receptor Current Charge Transfer 1–2 Weeks Post-injury

For synaptic charge transfer at 1–2 weeks post-CCI (Figure 4B and Table 4), a significant main effect of group was detected [2-Way RM ANOVA; *F*(2,37) = 6.73, *p* = 0.0032]. At baseline conditions, Ipsi-DGCs exhibit a synaptic charge transfer that is non-significantly reduced compared to Contra-DGCs (48% change; Tukey, *p* = 0.12) or Sham-DGCs (45% change; Tukey, *p* = 0.20). Markedly, in the presence of zolpidem, Ipsi-DGCs exhibit a synaptic charge transfer that is significantly decreased relative to Contra-DGCs (55% change; Tukey, *p* < 0.0001) and Sham-DGCs (44%; Tukey, *p* = 0.0088). The comparison of synaptic charge transfer between Sham- and Contra-DGCs revealed no significant differences before (Tukey, *p* = 0.97) or after zolpidem (Tukey, *p* = 0.23). A significant main effect of zolpidem was detected [2-Way RM ANOVA; *F*(1,37) = 129.8, *p* < 0.0001]. Zolpidem treatment significantly increased synaptic charge transfer in Ipsi-DGCs (78% change; Sidak, *p* = 0.0019), Contra-DGCs (106% change; Sidak, *p* < 0.0001), and Sham-DGCs (77% change; Sidak, *p* < 0.0001). The interaction between main effects was significant [2-Way RM ANOVA; *F*(2,37) = 9.041, *p* = 0.0006]. The application of zolpidem was therefore less effective in augmenting synaptic inhibition in Ipsi-DGCs, which is consistent with results from isolated DGCs taken from a rodent model of temporal lobe epilepsy (Brooks-Kayal et al., 1998). This is also despite the zolpidem-induced increase in sIPSC frequency observed in all groups (Figure 2).

### Tonic-Synaptic GABA Receptor Current Charge Transfer Ratio 1–2 Weeks Post-injury

For the ratio of charge transfer (I_TonicGABA_/I_SynapticGABA_) at 1–2 weeks post-CCI (Figure 4C and Table 4) a significant main effect of group was detected [2-Way RM ANOVA; *F*(2,37) = 14.97, *p* < 0.0001]. At baseline conditions, Ipsi-DGCs exhibit a charge transfer ratio that is non-significantly greater than Contra-DGCs (124% change; *p* = 0.19) and Sham-DGCs (103% change; Tukey, *p* = 0.25). In the presence of zolpidem, Ipsi-DGCs exhibit a ratio of charge transfer that is significantly greater than Contra-DGCs (499% change; Tukey, *p* < 0.0001) and Sham-DGCs (382% change; Tukey, *p* < 0.0001). The comparison of charge transfer ratio between Sham- and Contra-DGCs revealed no significant differences before (Tukey, *p* = 0.99) or after zolpidem (Tukey, *p* = 0.96). A main effect of zolpidem was detected [2-Way RM ANOVA; *F*(1,37) = 5.86, *p* = 0.021]. Zolpidem treatment significantly increased the charge transfer ratio in Ipsi-DGCs (125%; Sidak, *p* = 0.0001) whereas no significant change was detected in Contra-DGCs (−16% change; Sidak, *p* = 0.99) and Sham-DGCs (−6% change; Sidak, *p* > 0.99). No interaction of main effects was detected [2-Way RM ANOVA; *F*(2,37) = 7.65, *p* = 0.0017]. These results demonstrate that while tonic/synaptic GABAergic input is numerically imbalanced toward tonic GABAergic input early after CCI injury, the application of zolpidem amplifies this shift dramatically despite being a compound that is canonically characterized as selective for synaptic GABAergic input.

### Tonic GABA Receptor Charge Transfer 8–13 Weeks Post-injury

For the tonic charge transfer ratio at 8–13 weeks post-CCI (Figure 4D and Table 4), a significant main effect of group was detected [2-Way RM ANOVA; *F*(2,36) = 14.59, *p* = 0.0005]. At baseline conditions, Ipsi-DGCs exhibit tonic charge transfer that is not different than Contra-DGCs (Tukey, *p* = 0.94) and not different than Sham-DGCs (Tukey, *p* = 0.84); percent change not reported here since all groups are too similar. Markedly, in the presence of zolpidem, Ipsi-DGCs exhibit tonic charge transfer that is significantly greater than Contra-DGCs (77% change; Tukey, *p* = 0.015) and Sham-DGCs (74%; Tukey, *p* = 0.040). The comparison of tonic charge transfer between Sham- and Contra-DGCs revealed no significant differences before zolpidem (Tukey, *p* = 0.96) or after zolpidem (Tukey, *p* > 0.99). A significant main effect of zolpidem was also detected [2-Way RM ANOVA; *F*(1,36) = 2.064, *p* = 0.14]. Zolpidem treatment significantly increased tonic charge transfer in Ipsi-DGCs (84% change; Sidak, *p* < 0.0001), but non-significantly increased tonic charge transfer in Contra-DGCs (15% change; Sidak, *p* = 0.82) and Sham-DGCs (28% change; Sidak, *p* = 0.61). A significant interaction of main effects was detected [2-Way RM ANOVA; *F*(2,36) = 4.90, *p* = 0.013]. These results further emphasize the contribution of zolpidem to tonic inhibition of Ipsi-DGCs and that this effect is sustained after injury.

### Synaptic Charge Transfer for Phasic GABA Receptor Current 8–13 Weeks Post-injury

For synaptic charge transfer at 8–13 weeks post-CCI (Figure 4E and Table 4), there was no main effect of group [2-Way RM ANOVA; *F*(2,37) = 2.94, *p* = 0.066]. A significant main effect of zolpidem was detected [2-Way RM ANOVA; *F*(1,37) = 67.32, *p* < 0.0001]. Zolpidem treatment significantly increased synaptic charge transfer in Contra-DGCs (97% change; Sidak, *p* < 0.0001) and Sham-DGCs (81% change; Sidak, *p* < 0.0001), but was less impactful for Ipsi-DGCs (38%; Sidak, *p* = 0.0019). A significant interaction of the main effects was detected [2-Way RM ANOVA; *F*(2,37) = 7.40, *p* = 0.0020]. These results show that zolpidem application elevates synaptic input across groups similarly to earlier time points after CCI injury albeit with noticeable differences in the amount of augmentation of synaptic charge transfer in Ipsi-DGCs.

### Charge Transfer Ratio of I_TonicGABA_ and I_SynapticGABA_ 8–13 Weeks Post-injury

To better understand the balance of tonic and phasic GABAergic control in DGCs at later time points after CCI injury we compared the charge transfer ratio from these inhibitory sources (Figure 4F and Table 4). For charge transfer ratio at 8–13 weeks post-CCI, a significant main effect of group was detected [2-Way RM ANOVA; *F*(2,36) = 14.44, *p* < 0.0001]. At baseline, Ipsi-DGCs exhibit a ratio of charge transfer that is non-significantly greater than both Contra-DGCs (66% change; Tukey, *p* = 0.11) and Sham-DGCs (64% change; Tukey, *p* = 0.18). Markedly, in the presence of zolpidem, Ipsi-DGCs exhibit a charge transfer ratio that is significantly greater than Contra-DGCs (315% change; Tukey, *p* < 0.0001) and significantly greater than Sham-DGCs (251% change; Tukey, *p* < 0.0001). The comparison of charge transfer ratio between Sham and Contralateral DGCs revealed no significant differences before (Tukey, *p* = 0.99) or after zolpidem (Tukey, *p* = 0.95). The main effect of zolpidem was not significant [2-Way RM ANOVA; *F*(1,36) = 0.063, *p* = 0.80]. The interaction of main effects was significant [2-Way RM ANOVA; *F*(2,36) = 6.13, *p* = 0.0051]. Taken together with our observations 1–2 weeks after CCI injury, these results indicate that the relative balance of tonic/synaptic GABAergic input in Ipsi-DGCs remains shifted toward reliance on tonic inhibition at time points that correspond to numerous hippocampal circuit changes following injury. Additionally, application of zolpidem appears to further amplify this shift toward tonic inhibition, similar to earlier time points.

### Gene Expression of α_1_, α_2_, α_3_, and γ_2_ GABA Receptor Subunits in Dentate Gyrus 8–13 Weeks Post-injury

**FIGURE 5:**
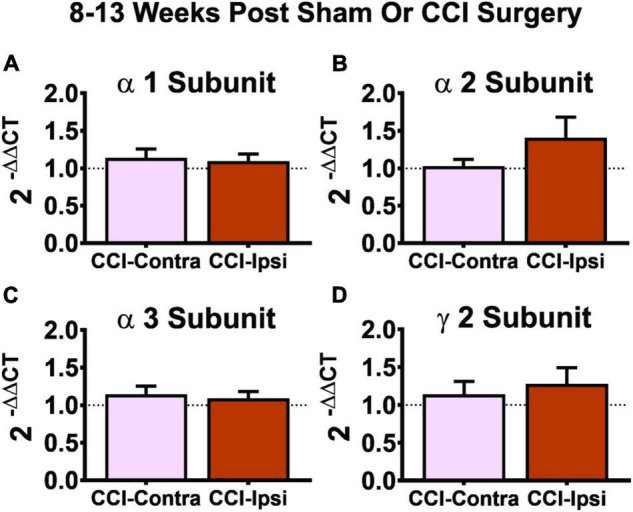
Gene expression of GABA_A_R subunits 8–13 weeks post-CCI. Comparisons of GABA_A_R subunits were made between the DG from the contralateral hemisphere (i.e., CCI-Contra) and ipsilateral hemisphere (i.e., CCI-Ipsi) relative to CCI injury. No effect was detected for the α_1_ subunit (**A**: CCI-Contra DG, *n* = 17; CCI-Ipsi DG, *n* = 19), α_2_ subunit (**B**: CCI-Contra DG, *n* = 8; CCI-Ipsi DG, *n* = 8), α_3_ subunit (**C**: CCI-Contra DG, *n* = 14; CCI-Ipsi DG, *n* = 16), or γ_2_ subunit (**D**: CCI-Contra DG, *n* = 9; CCI-Ipsi DG, *n* = 10). Delta cycle thresholds were used for statistical analysis (two-tailed *T*-tests) and data are presented as relative change using the 2^–ΔΔCT^ method (no change indicated by dashed line). Significance set as *p* ≤ 0.05.

The shift in influence of tonic vs. synaptic inhibition following CCI injury could be due to alterations in subunit expression of GABA_A_Rs in Ipsi-DGCs. To test this possibility, we assessed mRNA expression in the dorsal half of the isolated dentate gyrus and analyzed it for α_1_, α_2_, α_3_, and γ_2_ GABA_A_R subunit mRNA expression using samples from Contra- and Ipsi-DG at the 8–13 week time-point. No significant changes in mRNA expression of GABA_A_R α_1_, α_2_, α_3_, and γ_2_ subunit expression 8–13 weeks after CCI injury were detected (Figures 5A–D, respectively), suggesting that shifts in the functional influence of tonic vs. synaptic GABAergic input are not likely due to underlying changes in composition of GABA_A_Rs in Ipsi-DGCs.

## Discussion

The present study provides several key findings. First, tonic GABA_A_R signaling is spared in DGCs from the ipsilateral hemisphere of CCI-injured animals, an effect that occurs early after injury and persists for at least several weeks (Hunt et al., 2011; Boychuk et al., 2016; Butler et al., 2016, 2017; Zhu et al., 2019). Second, DGCs exhibit a zolpidem-sensitive I_TonicGABA_ that is markedly enhanced after CCI injury, which is surprising because zolpidem is traditionally considered to be a modifier of synaptic GABA_A_R signaling. Third, the proportion of charge transfer between baseline I_TonicGABA_ and I_SynapticGABA_ signaling (in DGCs) is non-significantly increased by CCI injury and profoundly enhanced by the combination of CCI injury and zolpidem. Fourth, GABA_A_R α_1_, α_2_, α_3_, and γ_2_ subunit gene expression is unchanged in Ipsi-DG relative to its contralateral counterpart at 8–13 weeks post-injury. These findings demonstrate the mechanistic disruption of GABA_A_R-dependent inhibition of DGCs at a cellular level following TBI. The results also demonstrate that zolpidem has surprising and powerful augmenting effects on I_TonicGABA_, and the proportion of charge transfer between tonic and synaptic GABA_A_R signaling in these cells, in a manner that is supra-additive with brain injury. These findings are consistent with a previous report that noted it is possible for GABA_A_R-modifying compounds such as midazolam and propofol to have differential responses of I_TonicGABA_ and I_SynapticGABA_ charge transfer in CA1 pyramidal neurons (Bai et al., 2001). Limitations of this study include how these cellular changes translate to post-injury hippocampus-dependent comorbidities such as posttraumatic epilepsy and hippocampal-dependent memory impairment and the potential for injury model-specific changes in GABAergic inhibition that we discuss in more detail below.

Altered GABAergic signaling is a common observation following brain insults, including TBI (Brooks-Kayal et al., 1998; Mtchedlishvili et al., 2010; Hunt et al., 2011; Pavlov et al., 2011; Raible et al., 2012, 2015; Boychuk et al., 2016; Butler et al., 2016, 2017; Frankowski et al., 2019; Zhu et al., 2019), and many treatment strategies attempt to rectify inhibitory control in disease through pharmaceutical targeting of the GABA_A_R’s, but with mixed results. Variation in how these different models of brain injury impact the components of inhibitory control (e.g., loss of GABAergic interneuron populations, changes in synaptic vs. extrasynaptic inhibition of target neurons, and excitation of surviving GABAergic interneurons) in the hippocampus is a potential source of these inconsistent outcomes. The CCI model has demonstrated reliable utility in understanding how severe TBI impacts hippocampal function, in particular because it closely reflects posttraumatic epilepsy outcomes in several key patient populations (Hunt et al., 2009, 2013), exhibits consistent loss of GABAergic interneuron populations (Lowenstein et al., 1992; Frankowski et al., 2019), and results in altered synaptic and tonic inhibition of DGCs in the hippocampus verified across multiple labs (Brooks-Kayal et al., 1998; Mtchedlishvili et al., 2010; Hunt et al., 2011; Pavlov et al., 2011; Raible et al., 2012, 2015; Boychuk et al., 2016; Butler et al., 2016, 2017; Frankowski et al., 2019; Zhu et al., 2019).

Inhibitory tone of DGCs in the hippocampus is directed by synaptic (phasic) and extrasynaptic (tonic) inhibition mediated by GABA_A_Rs (Chang et al., 1996; Mody, 2001; Brown et al., 2002; Stell et al., 2003; Wei et al., 2003; Semyanov et al., 2004; Farrant and Nusser, 2005; Mtchedlishvili and Kapur, 2006; Glykys et al., 2008). The mechanism(s) that alter GABA_A_R expression and function may be different depending on the injury model used, however, the work using CCI injury to model severe TBI has consistently demonstrated altered GABA_A_R function in DGCs ipsilateral to injury across multiple labs, highlighting the relevance of this dysfunction in TBI outcomes (Brooks-Kayal et al., 1998; Mtchedlishvili et al., 2010; Hunt et al., 2011; Pavlov et al., 2011; Raible et al., 2012, 2015; Boychuk et al., 2016; Butler et al., 2016, 2017; Frankowski et al., 2019; Zhu et al., 2019). One of the notable aspects of the present study, and our previous work describing δ subunit containing GABA_A_R’s, is that our observations of altered GABA_A_R function in DGCs ipsilateral to CCI injury were consistent at both early (1–2 weeks post-injury) and late (8–13 weeks post-injury) timepoints (Boychuk et al., 2016). This is particularly interesting given that many circuit alterations associated with posttraumatic epileptogenesis occur during the period between these timepoints, such as mossy fiber sprouting (Hunt et al., 2009, 2010; Butler et al., 2015) and *de novo* innervation and excitability changes of surviving GABAergic interneurons (Hunt et al., 2011; Butler et al., 2017), that parallel the change in inhibitory tone after CCI injury. These observations imply that while excitatory axon sprouting onto surviving interneurons may reflect a compensation for the loss of synaptic GABAergic input onto DGCs in the ipsilateral hippocampus, the preservation of tonic GABA_A_R signaling in DGCs also remains a critical mechanism to maintain hippocampal excitability and function.

In addition to molecular expression changes, multiple cell signaling mechanisms may affect GABA_A_R function in the DGCs ipsilateral to CCI injury (Butler et al., 2016; Becerra et al., 2021), and GABA transporters, chloride co-transporters and post-transcriptional mechanisms such as receptor trafficking and phosphorylation also contribute to I_TonicGABA_ function (Van Den Pol et al., 1996; Mody, 2001; Stell et al., 2003; Mody and Pearce, 2004; Rivera et al., 2004; Farrant and Nusser, 2005; Glykys and Mody, 2007; Glykys et al., 2008; Clarkson et al., 2010). Further, it is unclear whether such signaling processes occur exclusively within the injured DGCs or in combination with the reactive glia and other cells within the local injured environment. GABA-uptake mechanisms by astrocytes may serve important roles in these processes (Bai et al., 2001; Nusser and Mody, 2002; Semyanov et al., 2003; Clarkson et al., 2010). Recent work demonstrating improved behavioral outcomes following CCI injury after eliminating reactive microglia (Henry et al., 2020), as well as work showing the ability of overactive cell signaling cascades in microglia (e.g., mechanistic target of rapamycin, mTOR) to drive neuronal injury (Erlich et al., 2007) suggest important roles for non-neuronal cells in the underlying mechanisms that alter GABA_A_R function after TBI.

Although zolpidem has a 10-fold greater selectivity for α1 subunit containing GABA_A_R’s, this drug also interacts with GABA_A_R’s containing α2, α3 and α5 subunits (Hiu et al., 2016). Hippocampal DGCs express α_1_β_x_γ_2_ and α_4_β_x_δ GABA_A_R subunit combinations; other combinations are possible, and this is an active area of research. We have previously found that DGCs do not differ between contralateral and ipsilateral hemispheres of CCI-injured animals in their response to L655,708, a compound that preferentially agonizes α5 subunit containing GABA_A_Rs (Boychuk et al., 2016). We and others have also demonstrated a markedly reduced response to THIP or THDOC, agonists that are selective for GABA_A_Rs containing the δ subunit in Ipsi-DGCs, after CCI relative to control DGCs (Boychuk et al., 2016; Butler et al., 2016; Becerra et al., 2021). Despite our observed functional changes in GABA_A_R signaling, gene expression analyses here and previously (Boychuk et al., 2016) were unable to detect any expression changes in GABA_A_R α_1–5_, δ, or γ_2_ subunits ipsilateral to CCI at 8–13 weeks after injury. Immunohistochemical testing suggests a reduction in GABA_A_R δ and GABA_B_R β_2_ subunit immunoreactivity in DG about 2 weeks after CCI (Becerra et al., 2021), and western blot analyses detect a reduction in the α_1_ and γ_2_ GABA_A_R subunit protein expression 7 and 112 days after severe CCI (2.0 mm injury depth), whereas δ, β_2_ and β_3_ GABA_A_R subunits remained unchanged (Raible et al., 2015), and α_1_ GABA_A_R subunit mRNA expression is also reduced after severe CCI injury (Raible et al., 2015). Given that TBI results in a loss and/or disruption of synaptic GABA_A_R signaling, as indicated in our studies as a reduction in sIPSC frequency, but GABA_A_R mRNA and protein expression changes in this study and others have yielded few differences after CCI injury, an intriguing possibility is that post-synaptic populations of GABA_A_Rs (I_SynapticGABA_) can become orphaned and may thus be maintained after injury as extra-synaptic populations of GABA_A_Rs that could be zolpidem-sensitive and contribute to I_TonicGABA_. Mice with genetic deletion of the δ subunit exhibit a corresponding increase in γ_2_ subunit expression; thus a time-dependent inter-play between these subunits remains possible after CCI (Korpi et al., 2002). This line of inquiry requires further ultrastructural testing of GABA_A_Rs that mediate I_SynapticGABA_ and I_TonicGABA_ to more fully understand their location relative to the post-synaptic density (PSD), I_TonicGABA_’s dependence on GABA spill-over from the cleft, as well as time-course observation of PSD remodeling of GABAergic synapses during the evolution of the presynaptic injury caused by TBI. These findings suggest that subunit expression changes may develop in DGCs after severe CCI injury, but their contribution to the functional changes in GABA_A_R mediated responses reported here and elsewhere remains unclear.

The use of zolpidem to modulate GABA_A_R function highlights the importance of tonic inhibition of DGCs, especially in the hemisphere ipsilateral to injury. This is particularly interesting in light of recent work that implantation of progenitor GABAergic interneurons into the hippocampus following CCI injury is sufficient to restore synaptic inhibition and rescue behavioral impairments, including memory dysfunction, and reduce seizure susceptibility in these mice (Zhu et al., 2019). In particular, the influence of transplanted GABAergic interneurons on inhibitory synaptic input to DGCs suggests that these new GABAergic interneurons may restore the balance of synaptic and tonic inhibitory tone of the ipsilateral DGCs in a manner that is sufficient to correct the detrimental behavioral outcomes in these injured mice. It is unclear if these implanted interneurons integrate into the hippocampal circuitry similarly to those that migrate and integrate during neonatal development or if they receive *de novo* synaptic inputs similar to surviving GABAergic interneurons, as has been reported in this injury model and another model of epilepsy (Halabisky et al., 2010; Hunt et al., 2011; Butler et al., 2017). Regardless, a consistent theme in this study, and the literature for CCI, is the imbalance of inhibitory tone that develops in DGCs ipsilateral to CCI injury, as evidenced by an elevated reliance on tonic GABA_A_R-mediated inhibition. This tonic/synaptic GABA_A_R imbalance is observed early after injury, persists into timepoints when further axon reorganization and behavioral co-morbidities such as seizures develop in this model (Hunt et al., 2009, 2011, 2012, 2013; Halabisky et al., 2010; Butler et al., 2017). *De novo* excitatory synapse formation onto surviving GABAergic interneurons and other endogenous compensation mechanisms that develop during this time appear insufficient to modify this imbalance in inhibitory tone after injury. Further work is needed to understand if there is a way to enhance particular endogenous mechanisms to adjust this inhibitory balance or if exogenous methods, such as interneuron transplant, are the most effective strategies for improving the outcomes following TBI. The precise mechanism(s) by which DGC tonic GABA_A_R inhibition is spared and/or maintained after CCI is also critical to understand how to treat this imbalance and possibly learn how to better compensate for the loss of synaptic GABA_A_R signaling, which remains susceptible to damage after brain injury.

## Conclusion

This study highlights that GABAergic inhibition of DGCs in the ipsilateral hemisphere after CCI injury is biased toward tonic inhibition vs. synaptic inhibition (per the respective charge transfers of these modalities at both 1–2 and 8–13 weeks post-injury). The comprehensive view of cellular inhibition in this study provides several key insights into altered inhibitory control after TBI, but there remain limitations in our understanding of the underpinning mechanisms and downstream consequences of these cellular changes. The observation that application of zolpidem, a drug believed to preferentially target GABA_A_R’s associated with synaptic inhibition, further shifts this bias toward tonic inhibition raises some intriguing perspectives on our views of synaptic reorganization after brain injury, particularly as it highlights a potential influence of this drug in extrasynaptic transmission. Two diverging views regarding the role of more volume-based transmission include concepts that are either neuronal- or glial-centric. Neuronal-centric processes that have been postulated for these phenomena include changes in GABA_A_R subunits: reorganization, activity, trafficking and/or their localization, in a manner that may depend on phosphorylation of GABA_A_R subunits as well as changes in GABA_B_ receptor function. Glial-centric views may involve potential changes in synapse maintenance (Parkhurst et al., 2013; Rice et al., 2017), altered GABA transporter function (Yi and Hazell, 2006; Clarkson et al., 2010), and the need to reset “reactive” gene expression in glial cells after brain injury (Henry et al., 2020). Together, these diverging views demonstrate the great need to better understand how inhibitory synapses form, are maintained, and functionally contribute to phasic and tonic inhibition of target neurons in an integrative fashion involving many cell types and cell signals. Advancement in these research areas remains key to targeted intervention of disrupted excitation:inhibition balance found in a number of neurological diseases including posttraumatic epilepsy.

## Data Availability Statement

The raw data supporting the conclusions of this article will be made available by the authors, without undue reservation.

## Ethics Statement

The animal study was reviewed and approved by the University of Kentucky Animal Care and Use Committee.

## Author Contributions

All authors contributed to the conception and design of the work. JB, CB, KS, and MH performed the acquisition and analysis. All authors contributed to interpretation of the data, drafting and revision of the manuscript, and approved the final version to be published.

## Conflict of Interest

The authors declare that the research was conducted in the absence of any commercial or financial relationships that could be construed as a potential conflict of interest.

## Publisher’s Note

All claims expressed in this article are solely those of the authors and do not necessarily represent those of their affiliated organizations, or those of the publisher, the editors and the reviewers. Any product that may be evaluated in this article, or claim that may be made by its manufacturer, is not guaranteed or endorsed by the publisher.

## Abbreviations

GABA: gamma-aminobutyric acid
I_TonicGABA_: tonic GABA current
GABA_A_R: type A GABA receptor
CCI: controlled cortical impact
DG: dentate gyrus
TBI: traumatic brain injury
DGC: dentate granule cell
THIP: 4,5,6,7-tetrahydroisoxazolo[5,4-c]pyridine-3-ol hydrochloride
L655,708: 11,12,13,13a-tetrahydro-7-methoxy-9-oxo-9*H*-imidazo[1,5-*a*]pyrrolo[2,1-*c*][1,4]benzodiazepine-1-carboxylic acid, ethyl ester
qRT-PCR: quantitative real-time polymerase chain reaction
ACSF: artificial cerebrospinal fluid
sIPSCs: spontaneous inhibitory postsynaptic currents.
